# A move in the light direction

**DOI:** 10.7554/eLife.65360

**Published:** 2021-01-27

**Authors:** Eike M Wülfers, Franziska Schneider-Warme

**Affiliations:** 1 Institute for Experimental Cardiovascular Medicine, University Heart Center Freiburg – Bad KrozingenBad KrozingenGermany; 2 Faculty of Medicine, University of FreiburgFreiburgGermany

**Keywords:** cardiac optogenetics, spiral wave drift, defibrillation, sub-threshold illumination, Mouse

## Abstract

Computer simulations show how low-intensity illumination can be used to terminate cardiac arrhythmias.

**Related research article** Hussaini S, Venkatesan V, Biasci V, Romero Sepúlveda J, Quiñonez Uribe R, Sacconi L, Bub G, Richter C, Krinski V, Parlitz U, Majumder R, Luther S. 2021. Drift and termination of spiral waves in optogenetically modified cardiac tissue at sub-threshold illumination. *eLife* **10**:e59954. doi: 10.7554/eLife.59954

For a human heart to pump blood through our circulatory system, billions of muscle cells, called cardiomyocytes, must contract in a well-orchestrated manner. Ordered contraction is achieved via sequential electrical excitation of cells. Cardiomyocytes are excited when a sufficiently strong electric stimulus causes them to depolarize; they then remain in their activated state for some time; and, lastly, they return to their resting state, ready to be activated again. For normal heartbeats, electrical excitation originates from specialized pacemaker cells in the sinus node of the heart, which depolarize automatically. This rhythmically generated electrical signal then propagates along cardiomyocytes throughout the heart. A short time after depolarization, cardiomyocytes contract. Thus, rhythmical electrical activity leads to a regular heartbeat.

Cardiac arrhythmias can be caused by various factors – such as diseased cells and scar tissue – and they are associated with serious clinical conditions, including myocardial infarction, heart failure, and sudden cardiac death. One specific mechanism responsible for cardiac arrhythmia is so-called re-entrant electrical activity: this happens when the electrical impulse that excites cardiomyocytes does not terminate after all cells have been activated. Instead, due to pathological changes in cell or tissue properties, the excitation wave finds a way to circle back and re-activate cells out-of-turn. Under certain conditions, a self-sustaining ‘rotor’ can form, where electrical activity keeps circling in the heart for extended periods of time. While rotating, it emits spiral waves of electrical excitation that override the normal heart rhythm (Pandit and Jalife, 2013; Figure 1A).

**Figure 1.**
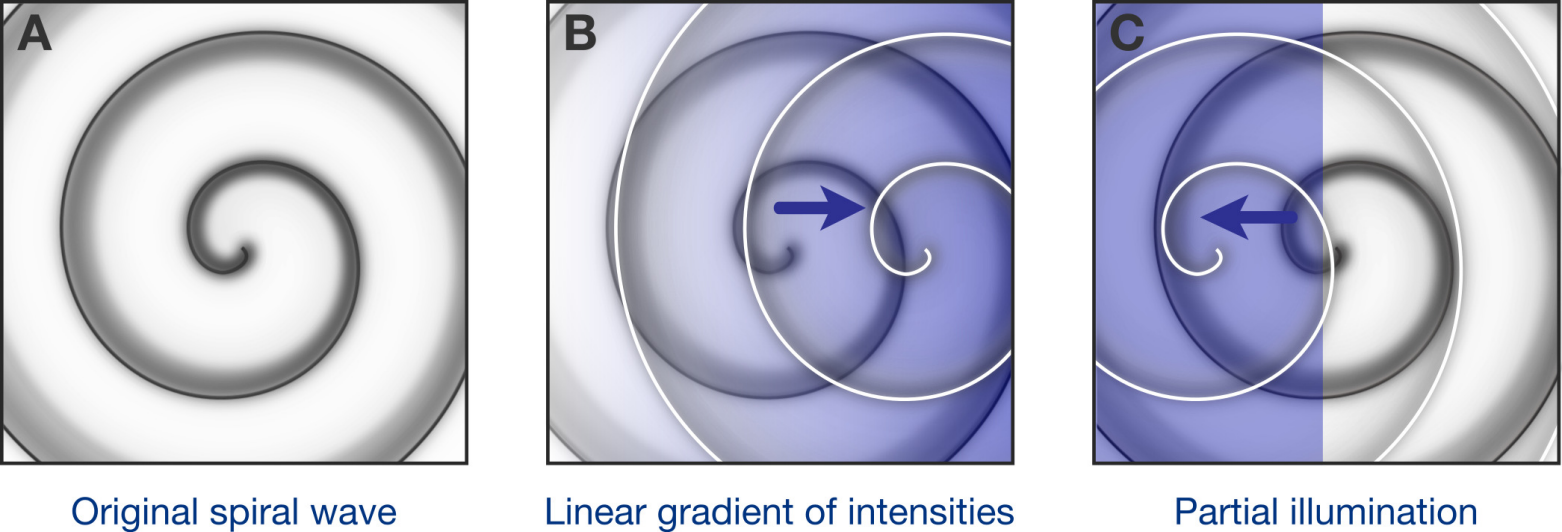
Optogenetic control of spiral waves. (**A**) Under certain conditions in the heart, spiral waves of electrical excitation are emitted by a ‘rotor’ of self-sustained electrical excitation. Hussaini et al. have used computer simulations to explore how a rotor in a two-dimensional model of heart tissue responds to various patterns of low-intensity illumination. (**B**) Upon illumination with a spatial gradient (here, the intensity increases from left to right), the rotor moves in the direction of higher light intensity. (**C**) Partial illumination of the model tissue with constant sub-threshold intensity light also causes the rotor to move towards the illuminated area.

One elegant way to study mechanisms of cardiac arrhythmias is optogenetics. In cardiac optogenetics, light-sensitive proteins are expressed in heart cells and are used to monitor or steer their electrical properties and function (Schneider-Warme, 2018; Zgierski-Johnston and Schneider-Warme, 2021). For example, blue light can be used to activate an ion-channel protein called channelrhodopsin-2, which results in the depolarization of cells. By activating this protein in cardiomyocytes, researchers have already successfully generated optical pacemakers (Arrenberg et al., 2010; Bruegmann et al., 2010) and conducted optical defibrillation in animal models (Bruegmann et al., 2018; Bruegmann et al., 2016; Crocini et al., 2016; Nyns et al., 2017). Now, in eLife, Stefan Luther and colleagues – including Sayedeh Hussaini as first author – report that low-intensity light may be used to steer rotors (Hussaini et al., 2021). Using a combination of cardiac optogenetics and computational modelling, they describe guiding rotors towards locations where re-entrant electrical activity is no longer possible, thereby terminating cardiac arrhythmias.

Computational modelling has long been used by cardiac researchers to explore how cellular (and sub-cellular) mechanisms act together in healthy or diseased heart tissue (Loewe et al., 2018). Hussaini et al. started with an established mathematical model of cardiomyocyte electrical activity (Petkova-Kirova et al., 2012) and added differential equations that describe light-activated currents mediated by channelrhodopsin-2 (Williams et al., 2013). A reaction-diffusion equation (the so-called monodomain model) was then used to couple multiple model cells in a two-dimensional tissue model.

Utilizing this model, the researchers – who are based at the Max Planck Institute for Dynamics and Self-Organization in Göttingen, the University of Göttingen and other institutions in Germany, France, Italy and Canada – first investigated the effect of illumination with intensities so low that the cardiomyocytes were slightly depolarized from their resting state, but were not excited. They found that increasing the intensity of such ‘sub-threshold’ illumination has two effects: it decreases the velocity at which electrical excitation is relayed from cell to cell, and it decreases the dominant frequency of spiral waves. Both results are in keeping with classically expected single-cell behaviour, but Hussaini et al. confirmed them in real tissue for the first time by performing *ex vivo* experiments with intact mouse hearts.

Next, Hussaini et al. simulated what could happen when using sub-threshold illumination with linearly graded light intensity. They found that the rotor – which previously had been stable – tended to move towards the part of the tissue where the light intensity was higher (Figure 1B). Moreover, the steeper the gradient, the faster the rotor moved. In another simulation, one region of the tissue was exposed to light of constant, low intensity, while the remaining tissue was not illuminated: here the rotor moved to the illuminated region (Figure 1C). Finally, they predicted that successive partial illuminations of the 2D tissue could be used to steer the rotor to parts of the tissue where the rotor could not continue to self-sustain.

Hussaini and colleagues propose a novel approach for the termination of cardiac arrhythmias using sub-threshold illumination. In fact, the underlying mechanism may have played a role in previous breakthroughs in cardiac optogenetics, such as the demonstration of optical defibrillation in animal models mentioned above. Further work is needed to verify effects of interventions proposed based on computational modelling, as hearts are characterized not only by a complex geometry, but also by tissue heterogeneities that rotors might become anchored to. Looking to the future, the obvious question is whether optogenetic modulation of cardiac electrophysiology, as described here, may ultimately be used to terminate arrhythmias in humans. Answering this question will require further research – it will be necessary, for example, to overcome the challenges associated with the expression of light-gated channels in cardiomyocytes, and with targeted light delivery. The work reported in this paper is undoubtedly an important step in the light direction.
